# Imaging hydroxyapatite in sub-retinal pigment epithelial deposits by fluorescence lifetime imaging microscopy with tetracycline staining

**DOI:** 10.1117/1.JBO.25.4.047001

**Published:** 2020-04-21

**Authors:** Henryk Szmacinski, Kavita Hegde, Hui-Hui Zeng, Katayoun Eslami, Adam C. Puche, Imre Lengyel, Richard B. Thompson

**Affiliations:** a University of Maryland School of Medicine, Department of Biochemistry and Molecular Biology, Baltimore, Maryland, United States; b Coppin State University, Department of Natural Sciences, Baltimore, United States; c University of Maryland School of Medicine, Anatomy and Neuroscience, Baltimore, Maryland, United States; d Queen’s University Belfast, Wellcome Wolfson Institute for Experimental Medicine, School of Medicine, Dentistry and Biomedical Science, Belfast, United Kingdom

**Keywords:** fluorescence lifetime imaging microscopy, age-related macular degeneration, drusen, tetracyclines, fluorescence ophthalmoscopy, hydroxyapatite, sub-retinal pigment epithelium deposits, retina

## Abstract

**Significance:** Recent evidence suggests that hydroxyapatite (HAP) in sub-retinal pigment epithelial (sub-RPE) deposits in aged human eyes may act to nucleate and contribute to their growth to clinically detectable size. Sub-RPE deposits such as drusen are clinical hallmarks of age-related macular degeneration (AMD), therefore enhanced and earlier detection is a clinical need. We found that tetracycline-family antibiotics, long known to stain HAP in teeth and bones, can also label the HAP in sub-RPE deposits. However, HAP-bound tetracycline fluorescence excitation and emission spectra overlap with the well-known autofluorescence of outer retinal tissues, making them difficult to resolve.

**Aim:** In this initial study, we sought to determine if the HAP-bound tetracyclines also exhibit enhanced fluorescence lifetimes, providing a useful difference in lifetime compared with the short lifetimes observed *in vivo* in the human retina by the pioneering work of Schweitzer, Zinkernagel, Hammer, and their colleagues, and thus a large enough effect size to resolve the HAP from background by fluorescence lifetime imaging.

**Approach:** We stained authentic HAP with tetracyclines and measured the lifetime(s) by phase fluorometry, and stained aged, fixed human cadaver retinas with drusen with selected tetracyclines and imaged them by fluorescence lifetime imaging microscopy (FLIM).

**Results:** We found that chlortetracycline and doxycycline exhibited substantial increase in fluorescence lifetime compared to the free antibiotics and the retinal background, and the drusen were easily resolvable from the retinal background in these specimens by FLIM.

**Conclusions:** These findings suggest that FLIM imaging of tetracycline (and potentially other molecules) binding to HAP could become a diagnostic tool for the development and progression of AMD.

## 1 Introduction

Age-related macular degeneration (AMD) remains the most common cause of blindness in elderly people living in the developed world, with over 10 million sufferers in the U.S. alone. A principal clinical hallmark of the disease is the development of deposits between the retinal pigment epithelium and the extracellular matrix termed Bruch’s membrane, especially in the macula;^1^^,^^2^ perhaps the best known of these are drusen. These deposits are widely held to contribute to retinal degeneration in part by slowing the flux of nutrients and oxygen to, and waste products from, the neurosensory retina.^3^

Recently, we discovered microscopic spherules of hydroxyapatite (HAP) in aging retinas between the Bruch’s membrane and the RPE.^4^ The spherules range from ∼0.5 to 10  μm in diameter, with the most frequent size of the distribution around 1.5  μm. They can be observed under the microscope even without substantial drusen material, which may represent the earliest stages of sub-retinal pigment epithelial (sub-RPE) deposit formation.^5^ Due to their micron size, detection of individual spherules by color ophthalmoscopy or fundus autofluorescence imaging is generally infeasible due to the limited resolution and a lack of contrast mechanisms. While the much larger HAP nodules (tens of microns in diameter) can evidently be detected by optical coherence tomography,^6^ the current state-of-the-art spectral domain optical coherence tomography with adaptive optics does not yet provide sufficient resolution^7^ to resolve individual spherules, although perhaps it will in the future. The spherules are frequently hollow, their shape and location make them distinct from the better-known calcification of Bruch’s membrane seen with von Kossa staining, and they are typically found coated with proteins previously identified in sub-RPE deposits, such as vitronectin, complement factor H, and amyloid beta.^8^ These findings led us to propose that the spherules may act to nucleate the growth of sub-RPE deposits; to date all sub-RPE deposits we have observed contain spherules. The likelihood that the spherules pre-date the ophthalmological detection of deposits suggested that they might serve as a useful biomarker for following the development of the earliest events leading to AMD. Apart from the spherules, recent evidence showed that a subset of HAP deposits that are larger (tens of micrometers in size) than the spherules and termed “nodules,” were associated with increased risk (odds ratio 6.4) of progression to advanced AMD within one year, providing good evidence for a direct link between HAP deposition and advanced AMD.^6^ Analysis of these nodules suggests that these are somewhat different from the small spherules but still largely composed of HAP, probably even in their cores, suggesting that they grew from spherules.^6^ Other HAP deposition is observed in the Bruch’s membrane.^6^

We found that the spherules could be imaged *in vitro* by fluorescence microscopy using several stains used for studying bone growth in animal models; the best performers had red- or infrared-fluorescing cyanine moieties conjugated to HAP-binding moieties.^9^^,^^10^ However, administering these stains in the eye is a challenge, they are not approved for use in humans, their toxicity and pharmacokinetics in humans are unknown, and any propensity for phototoxicity of these cyanine-based stains during fluorescence imaging of the retina remains unexplored. Among the stains tested were the legacy antibiotics of the tetracycline family, long known to bind HAP (like that in teeth) with accompanying fluorescence.^11^ However, there is substantial overlap of most tetracycline excitation and emission spectra with the well-known autofluorescence of the retina, which thus likely would interfere with imaging HAP stained with tetracyclines. Apart from this, the tetracyclines offer several prospective advantages: their safety, pharmacokinetics, absorption, distribution, metabolism, and excretion (ADME) are well known, and they mostly can be administered orally. However, we found that some tetracyclines exhibited not only increases in quantum yield upon binding to HAP but also substantial increases in lifetime, suggesting that HAP-bound tetracycline emission might be resolved from the retinal background using fluorescence lifetime imaging microscopy (FLIM), a technique wherein the contrast of the image reflects differences in fluorescence lifetime, instead of intensity.^12^ Thus this pilot study examined the ability of FLIM to resolve sub-RPE deposits in the aged human retina *in vitro* following tetracycline staining; some of these data appeared previously in an SPIE conference proceedings.^13^

## 2 Experimental Section

Tetracycline hydrochloride (CAS 64-75-5; Sigma-Aldrich), chlortetracycline (Cl-Tet) (57-62-5, Amresco), minocycline (10118-90-8, Sigma-Aldrich), and doxycycline HCl (24390-14-5, Sigma-Aldrich) were products of the indicated manufacturers and used without further purification. HAP beads [${\mathrm{Ca}}_{10}{\left(\mathrm{PO}4\right)}_{6}{\left(\mathrm{OH}\right)}_{2}$, Bio Rad] or tissue sections were stained with 0.1% solutions in pH 7.5 3-(N-Morpholino)propanesulfonic acid (MOPS)/NaCl buffer for 20 to 30 min then rinsed twice with pH 7.5 MOPS buffer. Postmortem human eyes were obtained from anonymous donors with prior informed consent. Retinas of fixed, enucleated eyes were dissected and flat-mounted with sclera side down. Since in these fixed specimens the neural retina was usually badly damaged, it was removed by forceps which partially removed the RPE, exposing the underlying drusen and Bruch’s membrane. Fluorescence lifetimes of tetracycline-labeled HAP beads suspended in mineral oil were measured on an ISS K2 multifrequency phase fluorometer with 409-nm laser diode or 442-nm HeCd laser excitation and Rose Bengal in ethanol as a standard, essentially as previously described.^14^ Fluorescence excitation and emission spectra were obtained on a Spectronics AB-2 fluorescence spectrophotometer. FLIM^12^ was performed on an ISS Alba with 443-nm excitation, 525±25-nm emission, using a 20× 0.4 NA objective and Coumarin 6 as a reference, essentially as previously described;^15^ the laser poorly excited doxycycline so retinal imaging was done only with CL-Tet labeling. FLIM image data (multifrequency phase and modulation data or time-resolved decays collected for individual pixels across the image plane) were fitted to discrete component models or lifetime distributions and displayed in the form of pre-exponential factor weighted average lifetimes $\tau_{\alpha }$, measured phases and modulations, or phasor plots^16^ using the manufacturer’s software. For single exponential decays collected in the frequency domain, the lifetime τ is a simple function of the phase shift φ or modulation m of the fluorescence emission with respect to the excitation at a given circular modulation frequency ω=2πf.^17^

tan φ=ωτ, (1)

$$m_{\text{emiss}}/m_{\mathrm{exc}}={\left(1+\omega^{2}\tau^{2}\right)}^{-1/2}.$$ (2)

For multiexponential decays [e.g., where $I(t)=\Sigma \alpha_{i}e^{-t/\tau }$)], the lifetimes and pre-exponential factors α are not simple functions of the phase and modulation, and thus one classically collects these data over a range of modulation frequencies and fits them to an assumed decay law in an iterative process similarly to that commonly used in the time domain to recover the αs and τs; see Lakowicz^18^ for a detailed description.

### 2.1 Data Analysis and Presentation

**Fig. 1:**
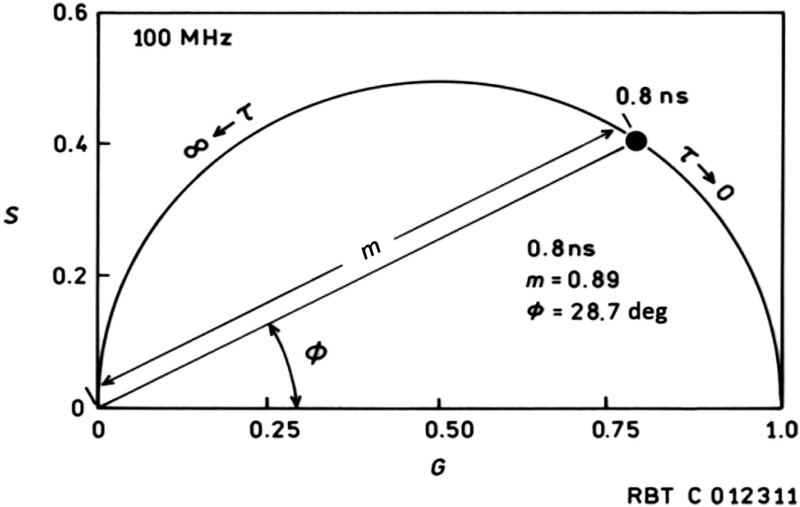
Phasor plot at 100-MHz modulation frequency; the black dot (•) indicates where a pixel with a 0.8-ns monoexponential decay would map: m=0.89, φ=28.7  deg.

A main goal in these experiments is to assess the utility of fluorescence lifetime as a contrast mechanism in imaging HAP in the retina, in view of the well-known autofluorescence of the retina whose spectral properties overlap those of the tetracyclines here. Thus we are more interested in resolving the emission of the stained HAP spherules from the background fluorescence, than necessarily determining the underlying lifetimes themselves. In particular, we wish to identify that subset of pixels having lifetime(s) corresponding to the stained HAP rather than accurately recovering the lifetime values *per se*. Thus, the lifetime dependence of the images was displayed in three different ways to assess which seemed to provide the best contrast in depicting the HAP spherules in the drusen: images color-coded for (1) the phase angle or modulation, (2) color-coded for the pre-exponential factor-weighted lifetime $\tau_{\alpha }$ as is widely used to display FLIM data, and (3) pixels exhibiting a particular subset of phase and modulations grouped using the phasor plot introduced by Redford and Clegg.^16^ For a phasor plot, the phase φ and modulation m of individual pixels at some modulation frequency are plotted in polar coordinates, where the length of the vector to that point from the origin is the modulation and the angle of the vector with the x axis is φ (Fig. 1).

In this system, a pixel exhibiting a monoexponential decay would map to the semicircular arc, whereas a pixel exhibiting a multiexponential decay would fall inside it. Pixels exhibiting similar decays would be closely grouped together on this plot, whereas an image composed of pixels having a range of decays would have points distributed accordingly over the phasor plot. In this case, the goal in using the FLIM approach is to create an image whose contrast (essentially) is based on differences in fluorescence decay, and in particular identify structures having different lifetimes compared with the background. The software enables us to highlight pixels in the fluorescence intensity image whose lifetime properties fall within a narrow range (indicated by a small circle) of points selected on the respective phasor plot.

## 3 Results

### 3.1 Fluorescence Lifetimes of HAP-Bound Tetracyclines

**Fig. 2:**
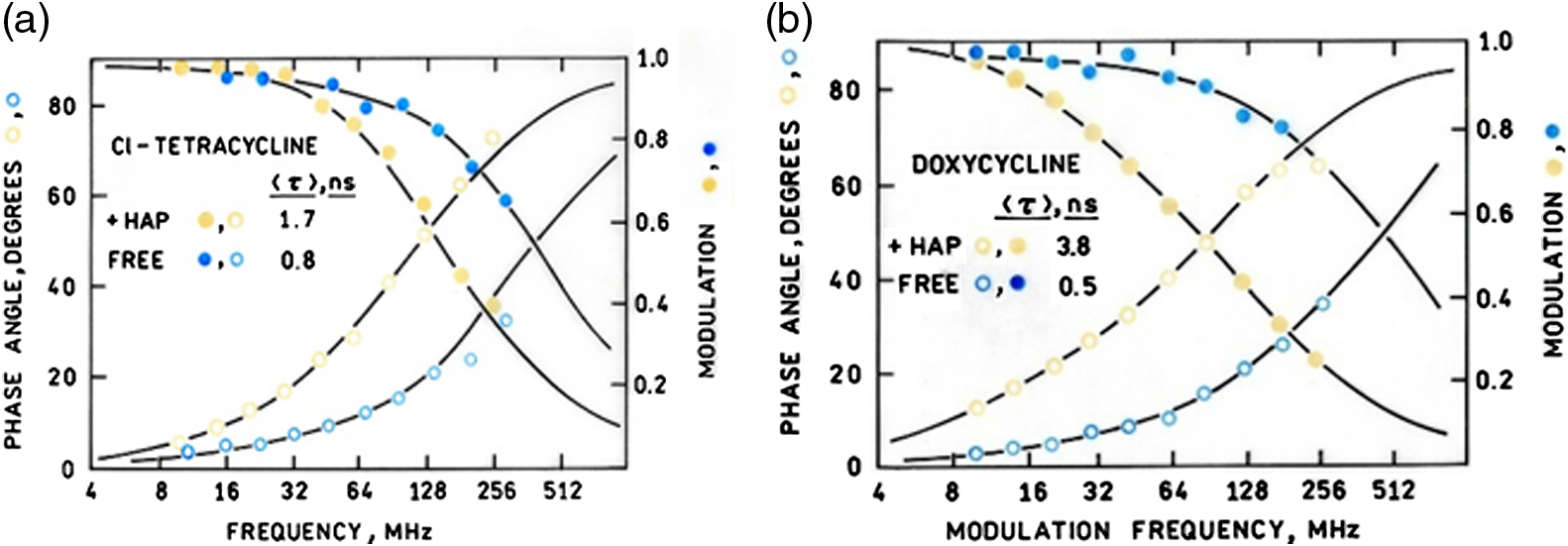
Frequency-dependent phase angles (open circles) and modulations (filled circles) for (a) Cl-Tet and (b) doxycycline bound to HAP (yellow) and free in solution (blue). The lines indicate the best two-component fits to the data.

Tetracyclines have long been known to stain bones and teeth with accompanying fluorescence, and it has been established that the tetracycline nucleus binds directly to the HAP moiety;^11^ indeed, a conjugate between tetracycline and a long wave fluorescent cyanine dye has proven highly selective as a bone stain.^10^ Many tetracyclines are weakly fluorescent in solution, and the apparent increase in brightness upon binding to HAP suggested that an increase in fluorescence quantum yield and lifetime might be occurring upon tetracycline binding to HAP. Thus authentic HAP was stained with each of the four tetracyclines above and the apparent lifetimes compared with those of the compounds dissolved in aqueous solution. Minocycline exhibited very weak fluorescence free and bound, and tetracycline exhibited negligible lifetime differences between the free and bound forms (results not shown). However, both doxycycline and Cl-Tet exhibited not only significant increase in intensity upon binding, but in average lifetime as well. Figure 2 depicts frequency-dependent phases and modulations for Cl-Tet and doxycycline free in solution and bound to HAP, with the best two-component fits to the data shown. Cl-Tet exhibited an increase in average lifetime τ from 0.8 to 1.7 ns upon HAP binding, and doxycycline exhibited an even more pronounced increase from ∼0.5 to 3.8 ns. The pioneering work of Schweitzer, Zinkernagel, Hammer, and their colleagues has shown that the average background autofluorescence lifetime of the healthy human retina *in vivo* is rather short, typically <0.5  ns,^19^^,^^20^ and suggests that the HAP-bound tetracycline emission could easily be resolved from the background on the basis of the former’s longer lifetime. Excitation and emission spectra of Cl-Tet and doxycycline in buffer and bound to HAP are displayed in Figs. S1 and S2 in the Supplementary Material, respectively. Since HAP-bound doxycycline is poorly excited with the laser line available on our FLIM, we chose to use Cl-Tet for staining experiments with retinal tissue. We note that the shorter excitation wavelengths necessary to excite bound doxycycline are less desirable due to increased phototoxicity, as well as greater attenuation and background fluorescence in the lens and vitreous.

### 3.2 Fluorescence Lifetime Microscopy of Aged Human Retina Labeled with Chlortetracycline

Retinas were flat-mounted, stained, and imaged by frequency-domain FLIM as described above. The variations in lifetime in the image field may be displayed as (1) maps of pre-exponential factors (α) and lifetimes (τ) made by fitting the frequency-dependent phases and modulations or time-dependent decays to assumed decay laws for individual pixels, or as (2) maps of average lifetimes calculated from fitted parameters, or as (3) maps of phase angles or modulations at a particular frequency, or as (4) maps of highlighted pixels having phase and modulation selected within some narrow range at some modulation frequency (phasor plot). Average lifetimes may be weighted either to pre-exponential factor α or fractional intensity f (for two components):

$$\langle \tau_{f}\rangle =f_{1}\tau_{1}+f_{2}\tau_{2},$$ (3)

$$\langle \tau_{\alpha }\rangle =\alpha_{1}\tau_{1}+\alpha_{2}\tau_{2}.$$ (4)

**Fig. 3:**
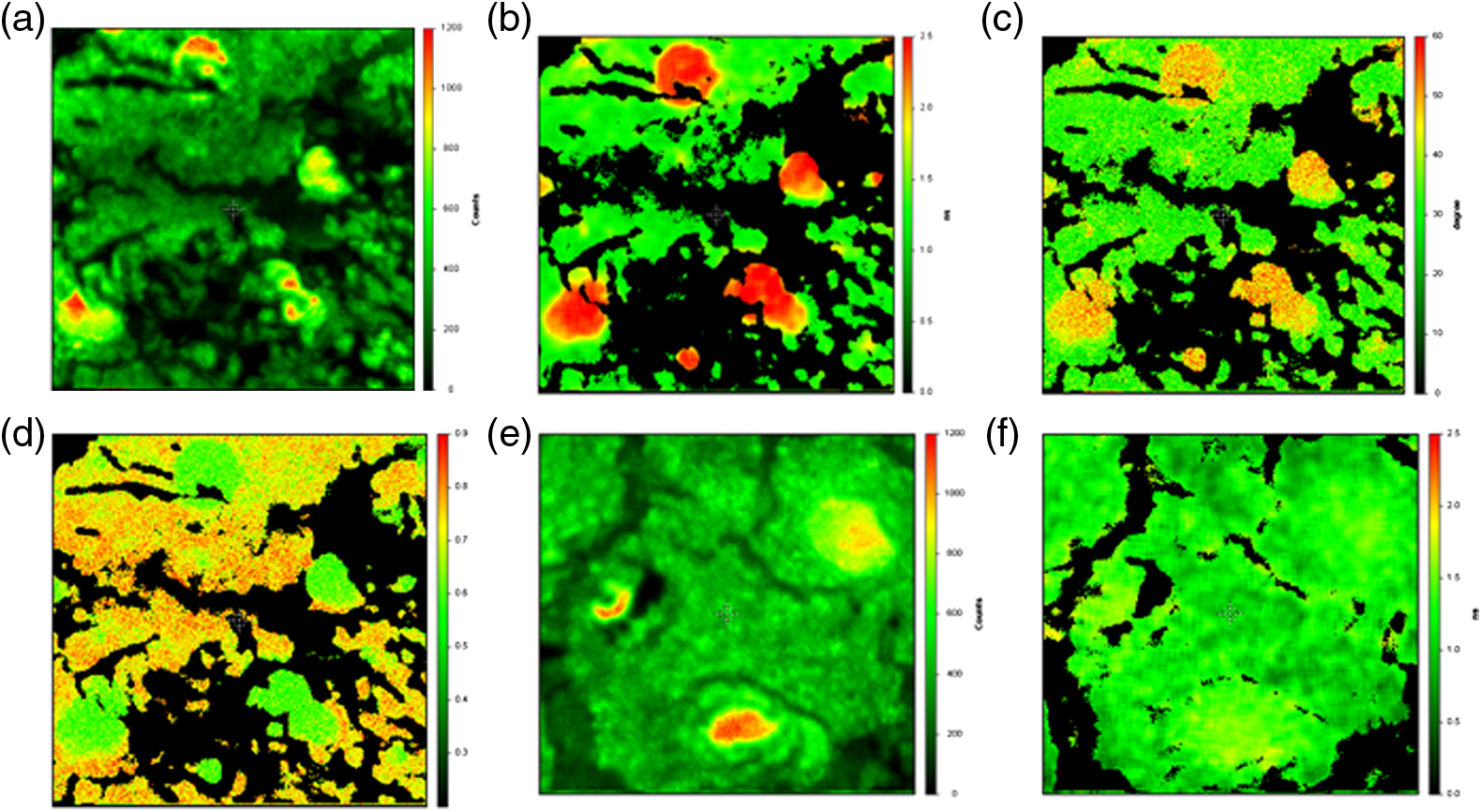
(a) A fluorescence intensity image of drusen (20× 0.4 NA objective, exc. 443 nm, emission 525±25  nm; $all\;panels\;240\;{\mu m}^{2}\;in\;size$) stained with Cl-Tet. (b) The same field with individual pixel values color-coded with pre-exponential value-weighted average lifetimes calculated according to Eq. (4) from the best two-component fitted parameters. Reddish areas on (b) corresponding to an average lifetime of ∼2.5  ns fitted to two components yielded $\tau_{1}=1.91\;\mathrm{ns}$, $\alpha_{1}=0.95$; $\tau_{2}=11.5\;\mathrm{ns}$, $\alpha_{2}=0.05$. (c) Color-coded phase angles and (d) modulations of the same tissue section are also shown. In (c), orange corresponds to roughly 50-deg phase shift and green corresponds to 30 deg, whereas in (d) green corresponds to roughly 60% modulation and orange to roughly 75%. (e) A fluorescence intensity image of an unstained retinal flat-mount fixed tissue section from the same donor at the same magnification, and (f) the FLIM image of the unstained section depicting color-coded pre-exponential weighted average lifetimes on the same color scale as (b). Green areas on (f) correspond to τ=1.43  ns, fit $\tau_{1}=0.89\;\mathrm{ns}$, $\alpha_{1}=0.87$; $\tau_{2}=4.9\;\mathrm{ns}$, $\alpha_{2}=0.13$.

This is illustrated for a series of fluorescence intensity and FLIM images of a flat-mounted Cl-Tet-stained fixed retina from a 94-year-old woman. Figure 3(a) depicts the fluorescence intensity image of the retinal field, and Fig. 3(b) depicts individual pixel lifetime data fitted to two components and the α-weighted average lifetimes calculated for those two components [Eq. (4)] are color-coded, with red being the longest lifetime.

The average lifetimes calculated for two components and color-coded on Fig. 3(b) show overtly better contrast with the background compared to the fluorescence intensity; this basic approach is how much FLIM data in the literature is depicted. We note that fitting these data to a two-component model is somewhat arbitrary, since there are likely to be several components and/or components exhibiting a distribution of lifetimes owing to subtle differences in their environments. Nevertheless, it is clear that the principal component 1 highlighted in red exhibits a lifetime (1.91 ns) close to that measured for Cl-Tet bound to synthetic HAP (1.7 ns) and comprises 95% of the emitters in those regions; we do not know if Cl-Tet binds to other compounds in the drusen and how that might affect Cl-Tet’s lifetime. We also note that fitting lifetime imaging data, either in the time or frequency domains, is a computational task that requires some time and thus is not available in real time.

If instead, the measured phase angles and modulation ratios at some suitable modulation frequency are color-coded for individual pixels in the same image, one obtains maps of the phases and modulations (shown for the same image field at 80-MHz modulation frequency) [Figs. 3(c) and 3(d)]. While the drusen are clearly apparent in Figs. 3(c) and 3(d) [orange in Fig. 3(c) and green in Fig. 3(d)], the differing color schemes make them seem to stand out less than the average lifetimes in Fig. 3(b). However, this may be mainly due to how the color differences are perceived by the viewer. This display method is different from the aforementioned display method in that it simply depicts the raw frequency-domain data without any computation, so it can be essentially used in real time, but reflects less information.

The drusen in the unstained fixed tissue section of drusen in Fig. 3(e) exhibit somewhat higher fluorescence intensity than the background, but without the Cl-Tet staining they exhibit somewhat less apparent contrast with background than those in the stained intensity image [Fig. 3(a)]. In striking contrast with the stained FLIM images, however, the average lifetime FLIM image of the unstained tissue [Fig. 3(f)] exhibits almost no contrast, and in this case the drusen are undiscernible.

**Fig. 4:**
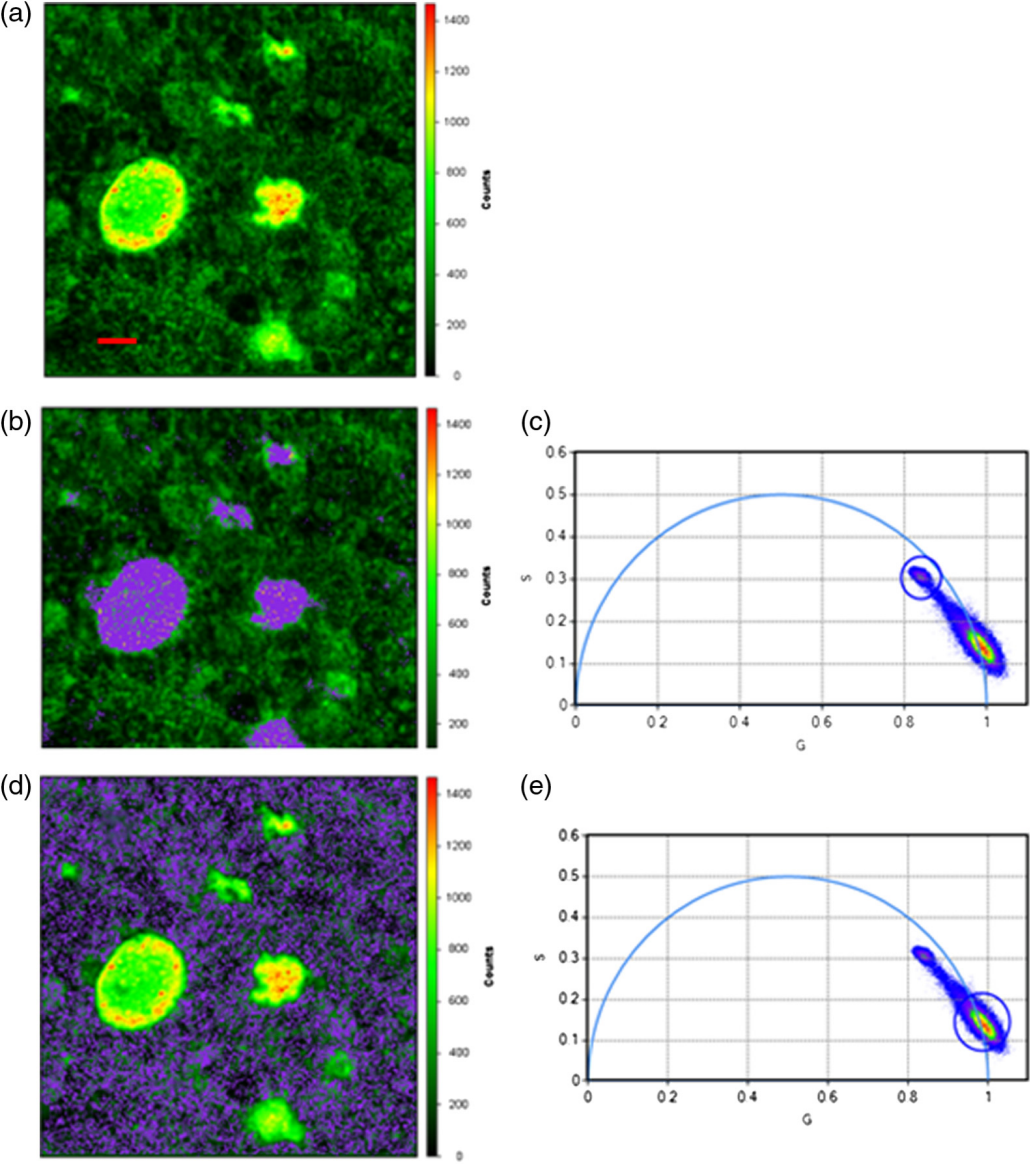
(a) Fluorescence intensity (excitation 443, emission 525±25  nm) image of flat-mounted retina of 94-year-old woman stained with Cl-Tet; scale bar=20  μm. (c) A phasor plot with circled points corresponding to pixels in the fluorescence image having ∼1.7  ns lifetime, which are highlighted in purple in the fluorescence image (b). (e) A phasor plot with the circle enclosing points corresponding to pixels with ∼0.6-ns lifetime, and (d) highlights pixels in the fluorescence image with the 0.6-ns lifetime in purple. The larger number of pixels mapped to points on the phasor plot with τ∼0.6  ns overlap strongly, which is indicated by the pseudocolors on the phasor plot.

The phasor plot described above enables one to highlight a subset of pixels in the intensity image based on their phases and modulations (Fig. 1). This is illustrated in Fig. 4, where panel (a) shows a Cl-Tet-stained fluorescence intensity image with prominent drusen, whereas in panel (b) a subset of pixels in the larger fluorescence image of Cl-Tet-stained retina having an average lifetime of 1.7 ns (corresponding to the HAP-bound Cl-Tet) are circled on the phasor plot (c) and highlighted in purple. In panel (d), pixels having a shorter average lifetime (0.6 ns) corresponding to the background are circled in panel (e) and highlighted in purple. The highlighted areas in panel (a) correspond to areas of brighter fluorescence identified as drusen. Therefore, this depiction also does not require a computational fitting process, but essentially uses the phasor plot to categorize all the pixels whose phase and modulation (and lifetime properties) fall within a given narrow range. Since no fitting is needed, it is also fast and has intrinsically high contrast (e.g., black and white).

**Fig. 5:**
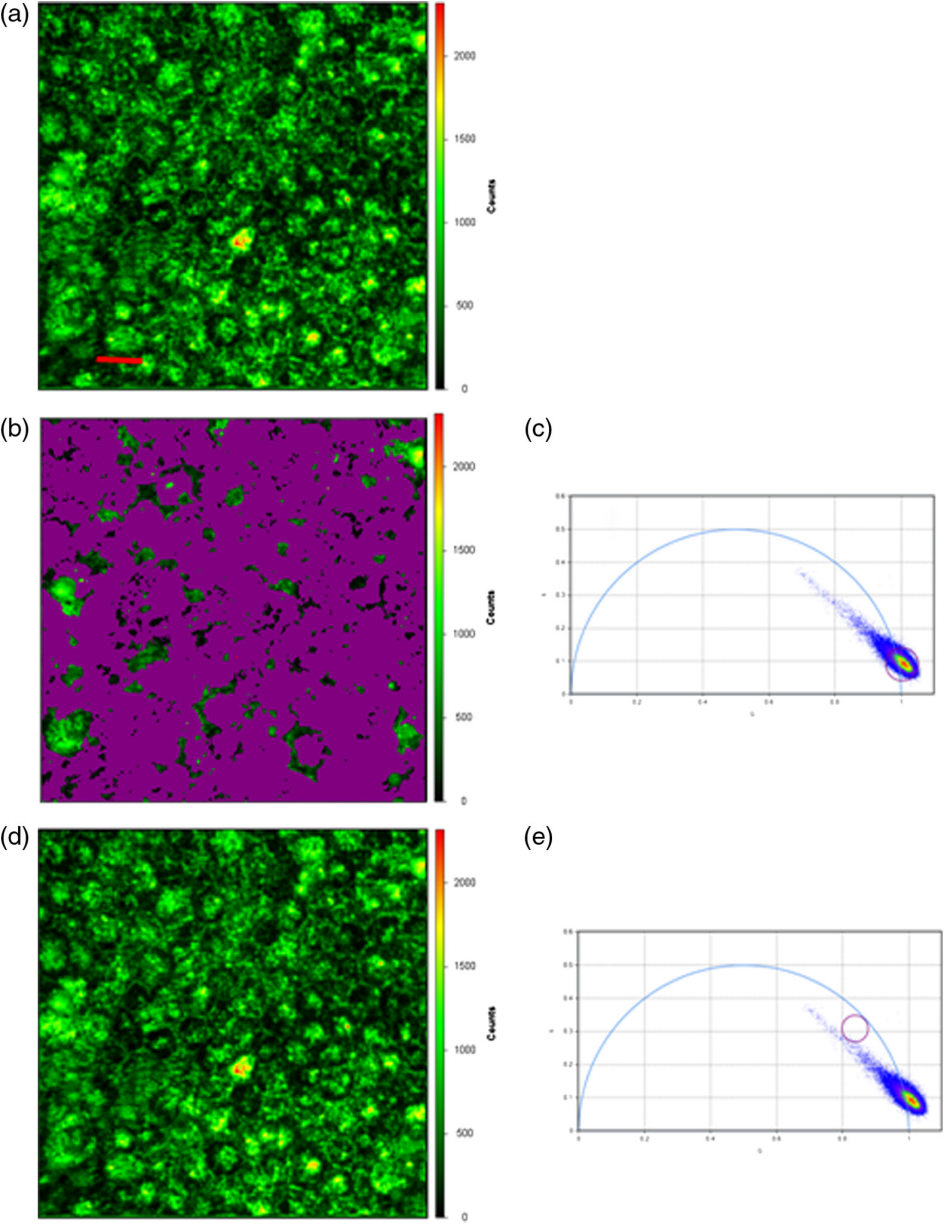
Unstained fixed retinal tissue from the same donor imaged under the same conditions with some RPE present, except higher laser power. (a) Fluorescence intensity image under same conditions as Fig. 3(a); scale bar=20  μm. (c) Phasor plot of all pixels with those with lifetime ∼0.5  ns encircled. (b) Pixels in purple having phase and modulation falling within the circle in (c), superimposed on the fluorescence intensity image. (e) Phasor plot of all pixels with lifetime ∼1.7  ns encircled. (d) Pixels highlighted in purple having phase and modulation falling within the circle superimposed on the fluorescence intensity image.

We also collected images of portions of the same unstained retina, which still contained areas of more or less intact RPE, and collected FLIM images under the same conditions as in Fig. 4, and analyzed them by use of the phasor plot; these images are collected in Fig. 5. As is apparent in the fluorescence intensity micrograph [Fig. 5(a)], the characteristic cobblestone phenotype of the RPE cells can still be seen in places, although it is no longer confluent. If the pixels with lifetimes of ∼0.5  ns are encircled on the phasor plot [Fig. 5(c)], then highlighted in purple and superimposed on the fluorescence intensity image [Fig. 5(b)], it can be seen that most of the pixels exhibit lifetimes in this short range; we note that the resolution of this FLIM for short lifetimes <500  ps is not as good due to the frequency range available. Finally, if we highlight pixels with a 1.7-ns lifetime on the phasor plot [Fig. 5(e)], we see that only a handful of widely spread pixels are highlighted in purple on the fluorescence image [Fig. 5(d)], indicating little 1.7-ns background (corresponding to Cl-Tet-stained HAP) is present. While this unstained, fixed tissue does not exhibit precisely the same background lifetime as observed *in vivo* by fluorescence lifetime ophthalmoscopy, it is clear that even with RPE cells present there is little emission with lifetimes corresponding to the emission of Cl-Tet-stained HAP that might potentially interfere.

## 4 Discussion

In this individual, the Cl-Tet-labeled drusen can be distinguished from the background on the basis of intensity [Figs. 3(a) and 4], but the well-known overlap between the Cl-Tet emission and the autofluorescence of the retina (especially the RPE) make identification of some deposits and HAP challenging. However, it is apparent from Figs. 3(b)–3(d) and 4 that the drusen can be resolved from the autofluorescence “background” of the retina in these fixed specimens by the differences in lifetime. The lifetime properties of retinal tissue from the same donor with RPE cells remaining are also clearly resolvable from those of Cl-Tet-stained HAP (Fig. 5). The lifetime of the background fluorescence observed here differs from (is slightly longer than) that observed by Dysli et al.^20^ in healthy retinas, but this might be due to the fixation of the samples used in our experiments, and the modest resolution of such short decays at the modulation frequencies available in our instrument. However, the lifetime differences between drusen and background are still large enough to clearly distinguish them from each other. We note that fluorescent stains developed for studying bone growth (Bone Tag: LiCor; OsteoSense: Thermo-Fisher) absorb and emit at significantly longer wavelengths such that the background autofluorescence is minimal, but the ADME, pharmacokinetics, toxicity, and particularly phototoxicity are unknown, and thus they are not yet approved for human use, and administration is a challenge.

Among the advantages of the phasor plot (Figs. 4 and 5) over plots of phase and modulation data (Fig. 3, Panels C and D) is that it combines the phase and modulation information (at one frequency) instead of leaving them separate. While it does not include the complete decay, the frequency of the phasor plot can be chosen to optimize the differences in how pixels with particular lifetime properties map. On the other hand, the average lifetime essentially combines the entire decay in a single parameter, and pre-exponential factor or fractional intensity-weighted averages may be chosen to emphasize short or long decays. We also note that the phase angles may be collected in microseconds,^21^ suggesting that data collection at least in the frequency domain is potentially very fast. We hasten to add that other time-domain approaches, including time-correlated single photon counting and the rapid approaches developed in the Grundfest and Marcu labs, may offer advantages, especially in clinical applications.

As the HAP-bound lifetime changes of Cl-Tet and doxycycline differ substantially from the measured retinal background lifetimes measured by others*in vivo*, we expect to resolve the stained spherules on the basis of lifetime in the living eye in the future. There are decades of experience with the tetracyclines in humans, and their ADME in humans is well enough understood to permit their use in children;^22^ moreover, the pharmacokinetics of doxycycline and Cl-Tet in humans are well known, and they are likely to be effective staining the retina when administered orally.^23^^,^^24^ Importantly, these parameters are also known for these tetracyclines in macaques, the best animal models found to date for studying the development of AMD.^25^^–^^29^ FLIM has now been adapted to an ophthalmoscope for use in humans;^19^^,^^20^ therefore, we suggest that tetracycline-based FLIM could become a potentially valuable approach for detection of spherules as an early marker for the development of sub-RPE deposits. The recent discovery^6^ that larger HAP deposits in the eye (tens of microns, termed nodules) confer high risk for progression to advanced AMD within one year further supports our contention that HAP spherules may be a useful biomarker for early detection of AMD. Our approach not only allows early detection but also assessing the progress of the disease to initiate treatment before irreversible vision loss occurs, which is not currently feasible. We believe that most people would agree that intervening early in the development of AMD and other diseases before vision is lost irreversibly is a desirable strategy. The ability to monitor early progression additionally will facilitate testing of innovative treatment strategies for AMD currently under development. Finally, the development of imaging approaches for calcification such as these may also be useful for other diseases.
