# Supplementary material for: Imaging hydroxyapatite in sub-retinal pigment epithelial deposits by fluorescence lifetime imaging microscopy with tetracycline staining
Source: J Biomed Opt. 2020 Apr 21;25(4):047001. doi: 10.1117/1.JBO.25.4.047001 (PMC7171513; doi:10.1117/1.JBO.25.4.047001)
Supplement: Supplementary file 1 [file JBO_025_047001_SD001.pdf]

We measured excitation and emission spectra of chlortetracycline (Cl-Tet, Figure S-1) and Doxycycline (Figure S-2) free in physiological saline buffer (dashed lines) and bound to hydroxyapatite beads suspended in mineral oil (solid lines); the normalized spectra are depicted below:

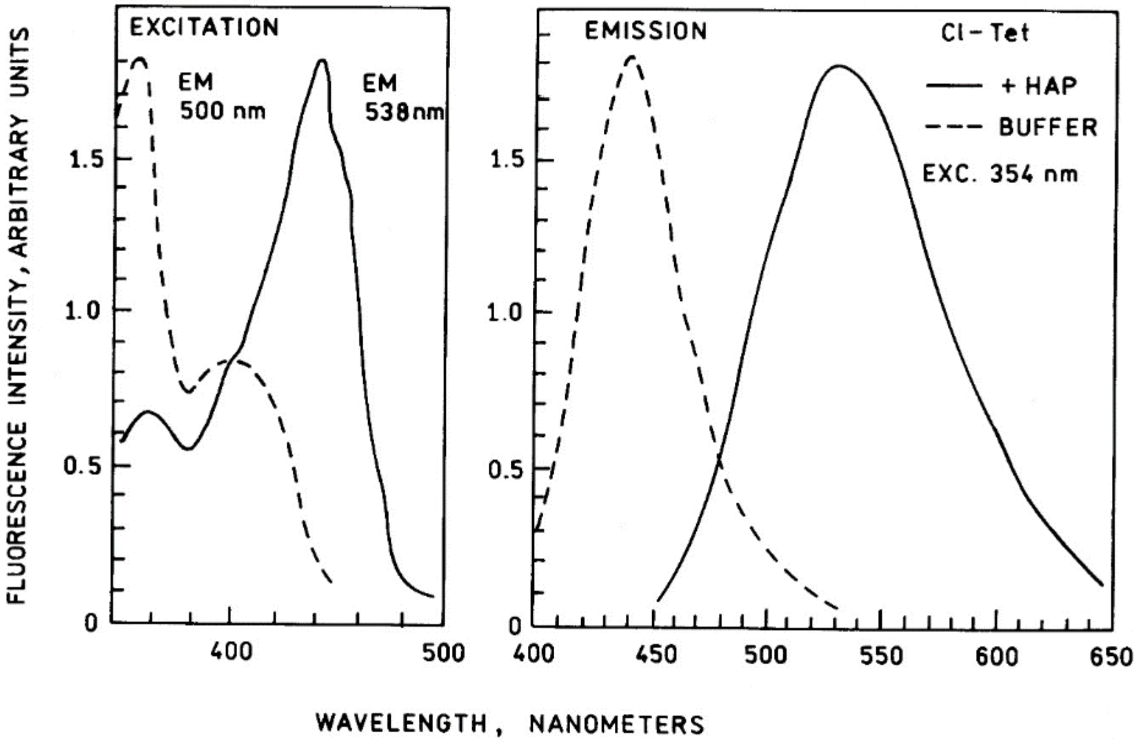

Figure S-1: Excitation spectra (left panel; Cl-Tet bound to HAP [solid line, emission wavelength 538 nm] and dissolved in buffer [dashed line, emission at 500 nm]) and emission spectra with

excitation at 354 nm (right panel; Cl-Tet bound to HAP [solid line,] and dissolved in buffer [dashed line]).

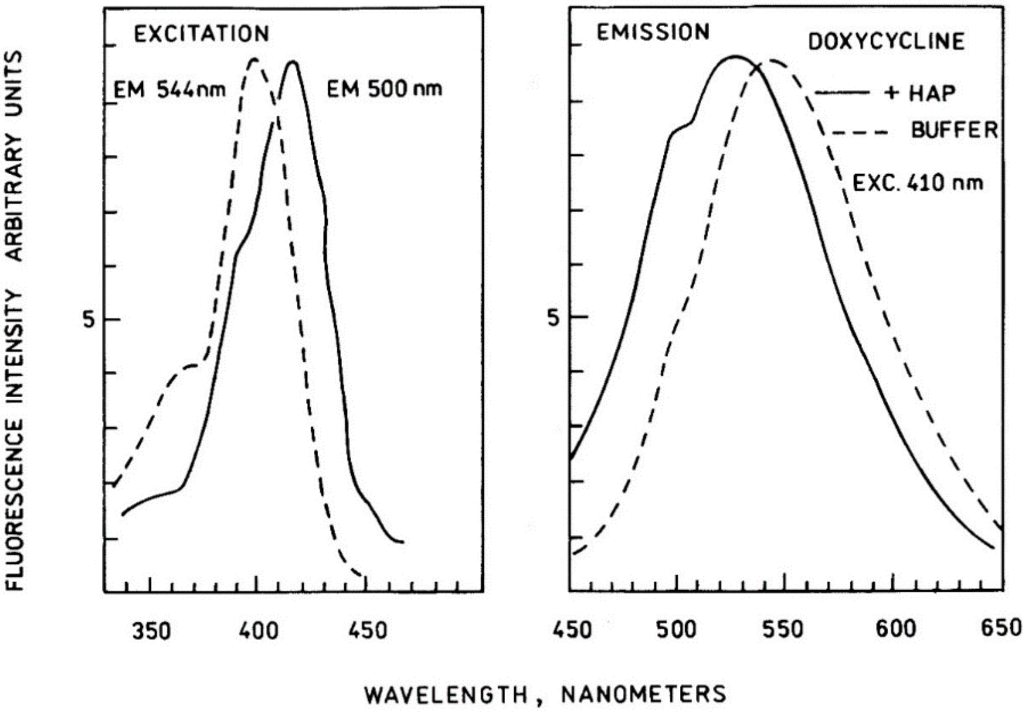

Figure S-2: Excitation spectra (left panel; doxycycline bound to HAP [solid line, emission wavelength 500 nm] and dissolved in buffer [dashed line, emission at 544 nm]) and emission spectra with excitation at 410 nm (right panel; Cl-Tet bound to HAP [solid line,] and dissolved in buffer [dashed line]).

The spectra make evident the slight shifts in emission and excitation of doxycycline upon binding to HAP, as well as the larger ones apparent for Cl-Tet. Interestingly, the Stokes' shift of the Doxycycline shrinks upon binding, with the excitation and emission exhibiting red and blue shifts, respectively, upon binding. By contrast, the excitation and emission of Cl-Tet both red shift upon binding to HAP; in this case the emission shifts substantially and the Stokes' shift is much larger for the bound Cl-Tet than Doxycycline.
